# Motivations and Challenges for Adoption of Organic Grain Production: A Qualitative Study of Iowa Organic Farmers

**DOI:** 10.3390/foods11213512

**Published:** 2022-11-04

**Authors:** Guang Han, Nancy Grudens-Schuck

**Affiliations:** 1 College of Humanities and Social Development, Nanjing Agricultural University, Nanjing 210095, China; 2 Institute of Regional Agricultural Research, Nanjing Agricultural University, Nanjing 210095, China; 3 Department of Agricultural Education and Studies, Iowa State University, Ames, IA 50011, USA

**Keywords:** organic grain, sustainable food systems, adoption, motivations, challenges, farmers, qualitative study

## Abstract

Organic grains are essential for the organic food industry. In the U.S., low adoption of organic grain farming has constrained further development of the organic food sector. Organic food industry stakeholders have appealed to producers to increase domestic organic grain production. The U.S. federal government supports research and extension education regarding organic farming. In this context, there is a need for both agricultural researchers and extension professionals to further (1) examine the factors that motivate farmers to adopt organic grain farming and (2) identify the challenges that hinder farmers’ adoption of organic grain farming. We conducted 17 in-depth interviews with organic grain farmers in Iowa, USA. By applying multiple social-behavioral theories as part of the analysis and comparing interview results with the literature, we gained insight into the ways in which farmers formed adoption motivations, and we captured the dynamics of the motivations. We specifically identified challenges to adoption that were associated with organic farming operation and management, organic market accessibility, information and inputs availability, social tension, and level of support from the government. These findings shed light on the ways in which farmers’ adoption challenges have evolved with institutional, ecological, and technological changes over time and how contemporary research and extension may encourage adoption.

## 1. Introduction

Organic grains are essential organic food ingredients for human consumption and animal feed, yet the adoption of organic production practices for grain remains low. For instance, organic corn accounted for 0.38% of total corn harvested acreage in the U.S., 0.18% of soybean production was organic, and 1.16% of wheat acreage was organic [1,2]. High demand for organic grain has led stakeholders, including government officials, organic food processors, and organic producer associations, to appeal for increases in the production of organic grain by U.S. farmers [3,4,5,6]. However, the low adoption of organic grain production has raised additional questions regarding the interactions of motivations, barriers, and challenges that contribute to adoption. Studies conducted from the 1970s onward have examined farmers’ motivations for adopting organic farming of a range of crops in a variety of regions. Many studies also examined barriers but documented experiences mainly for small-scale diversified organic farms (e.g., fruit, vegetable, and livestock). This study focused on the adoption of organic grain farming in the U.S. Midwest.

### 1.1. Productivity and Demand

Consumer demand for organic foods continues to grow and requires a large supply of organic grains for food processing and animal feed [7]. However, demand has not been satisfied by U.S. organic grain production alone [8]. Charles [3] and Greene et al. [4] argued that recurring shortages of organic grain are due to the low adoption of organic grain production practices by U.S. farmers. Roseboro [6] reported that organic food processors had been occasionally forced to cut their product lines due to an insufficient supply of organic ingredients.

According to USDA National Organic Program (NOP) regulations, to obtain certification for organic meat, eggs, and dairy products, organic livestock producers must feed livestock with organic grains and hay [9]. Digiacomo and King’s study [10] showed that organic livestock farmers reported insufficient supplies of organic feed, as well as a lack of affordable grains and consistent availability. Sierra et al. [11] argued that under-supply and poor access to organic feed became barriers for conventional livestock producers entering the organic sector and a causal factor for organic livestock producers who eventually deregistered from an organic certification program. A key reason is that organic grain farming adoption across commodities requires farmers to make systemic changes in their operations [12,13]. Organic farming generally prohibits synthetic chemicals in crop production, and farmers must learn new techniques, including cultivation, crop rotation, and use of biological pest control to minimize risks to productivity and profit [12,14,15]. Farmers who intend to certify products as organic are required to undergo a three-year transitional period. The transition process has been demonstrated to increase farmers’ financial risks in some cases [12,16]. 

### 1.2. Environmental, Social, and Economic Impacts

Insufficient adoption also restricts the scope of positive impacts that organic farming may provide to the environment, economy, and society. In the U.S. Corn Belt, surface water that moves into the Mississippi River watershed is contaminated with phosphorus and nitrogen from agricultural fertilizers and sediment. As contaminants accumulate, they contribute incrementally to the hypoxic “dead zone” in the Gulf of Mexico [17,18]. In two separate studies, Cambardella et al. [19] and Hole et al. [20] showed organic farming systems could mitigate surface water pollution by reducing nitrate leaching and the amount of pesticide residue in the environment. Wider adoption of organic farming in the Corn Belt could help address these and other environmental issues. Organic farming also has the potential to become an economically sound farming system that enhances profitability by lowering production costs, securing price premiums, and achieving yields comparable to conventional production systems [21,22,23]. Organic farming can boost rural communities by increasing household income, generating employment, retaining output values in the local economy, and lowering the county-level poverty rate [24,25].

Organic livestock producers, food processors, and consumers together have called for increased domestic organic grain production [6,26,27]. However, it falls to the people who manage farms and decide on the production practices they will use. Producers have complex motivations, needs, and situations for decisions they make for themselves, their farms, and their family situations. The federal government has progressively issued more supportive policies for organic agriculture in the Farm Bill legislation, from the 1985 Farm Bill to the 2018 Farm Bill [4,28,29]. The USDA allocated funding to support land-grant universities and agricultural non-profit organizations to develop and deliver extension education and research programs to promote organic farming and address related issues [29,30]. To promote organic farming, Trout et al. [31] recommended that outreach and extension programs should stimulate farmers’ learning to accelerate organic farming conversion. They, thus, suggest that agricultural extension professionals and organic agriculture specialists pinpoint farmers’ motivations for adoption and identify challenges that impede farmers’ adoption of organic grain farming.

Farmers’ decisions related to the adoption of organic grain farming are intricate, however, because aspects of a farming system interact with social, economic, institutional, and biophysical conditions. Motivations for adopting organic farming broadly include economic incentives, environmental benefits, human and livestock health, and ideological as well as religious beliefs [12,24,32,33,34,35,36,37,38,39,40,41,42]. Common barriers to adopting organic farming have historically included a lack of information, limited inputs, marketing problems, difficulties with weeds and pest control, constricted land tenure, unfavorable policies, and negative social pressures [12,15,16,34,35,36,41,42,43,44,45,46].

In the U.S., most existing studies on organic farming adoption sampled producers from a mixture of different commodity types and heavily sampled producers of specialty crops (fruit and vegetables) and livestock [12,16,32,35,36,38,47]. Fewer studies focused on grain farmers [40,48,49]. Lockeretz [44] cautioned that it would oversimplify the story of the production and marketing of organic foods to treat organic farmers of all commodity types as a single, unified group. Anderson et al. [32] also recognized that given the large differences in growing conditions, management practices, and market structure, studies on fruit and vegetable producers could not represent grain farmers. 

To deepen understanding of farmers’ organic grain farming adoption decisions and provide agricultural extension educators and organic specialists with key information to develop and deliver effective extension programs, this study aimed to answer two research questions:

1. What are the motivations for farmers to adopt organic grain farming?
2. What are the challenges faced by farmers in adopting organic grain farming?

## 2. Theoretical Models

### 2.1. Motivation Theory

Motivation is a common term that has a plain language meaning, but the study applied a theoretical body of knowledge to bring more rigor to the examination of motivations related to adoption levels and reasoning related to organic practices in grain farming. Motivation in the psychological literature is defined as “the psychological process that initiates, guides, and maintains human behavior” [50] (p. 1204). Although many authors describe farmer behavior as relying on a general description of motivation, knowing the structure of motivations can facilitate deciphering farmers’ interwoven decisions regarding decisions related to farming [51,52,53,54]. Self-determination theory (SDT), a well-regarded theory of motivation, distinguishes motivations based on reasons and goals of action: intrinsic motivation and extrinsic motivation [55]. Extrinsic motivation refers to “doing something because it leads to a separable outcome” [55] (p. 55). Intrinsic motivation refers to “doing of an activity for its inherent satisfactions rather than for some separable consequence” [55] (p. 56). SDT proposes a self-determination continuum that internalizes extrinsic motivations into intrinsic motivations through four regulation procedures: “external regulation” (e.g., a reward), “introjected regulation” (e.g., self-esteem or feeling of worth), “identified regulation” (e.g., goals or importance), and “integrated regulation” (e.g., personal beliefs and values) [56] (p. 16). There is no “best” type of motivation, and the array must be explained in context.

Employing this theory, scholars have typically found farmers’ intrinsic motivations are the primary motivations for adopting sustainable farming practices, including land stewardship beliefs, biodiversity values, animal welfare ethics, public health concerns, and community resilience [52,53,54]. In comparison, extrinsic motivations tend to be secondary motivations for environmental farming behaviors, which include special financial incentives, profit maximization, farming viability, and regulatory compliance [51,53,54]. These empirical examples helped to create the interview guide of organic farmers’ motivations in this study.

### 2.2. Behavioral Theory

A second theory focused less on inner reasoning and more on behavior and action. The theory of planned behavior (TPB) states people’s behavioral intention to perform a behavior is motivated by attitudes toward the behavior and subjective norms but controlled by ability [57,58]. Thus, TPB bridges the motivating factors and behavioral challenges for this study. The TPB is often paired with the Diffusion of Innovations (DOI) theory by Rogers [59], and organic farming is an innovation based on knowledge and information, as defined by DOI [13]. Attitude toward the behavior is “the degree to which a person has a favorable or unfavorable evaluation or appraisal of the behavior” [57] (p. 188). Farmers’ attitudes towards organic farming can be manifested by individuals’ perceptions regarding organic farming’s relative advantages and observability, which can be traced back to the theory of diffusion of innovation [59]. 

Subjective norm refers to “the perceived social pressure to perform or not to perform the behavior” [57] (p. 188). Subjective norm seems to connect well with the concept of “compatibility” in DOI. Rogers [59] indicated if an innovation is not compatible with prevalent social norms in a social system, the innovation will not be rapidly adopted. The low adoption rate and slow growth of organic farming in the U.S. may be attributed to its “alternative agricultural paradigm”, which conflicts with the American mainstream agricultural production paradigm in multiple dimensions [60]. 

Behavioral control is “perceived ease or difficulty of performing the behavior” [57] (p. 188). Individuals with strong behavioral control are those who have the necessary resources, knowledge, skills, and opportunities to perform a behavior [57]. Conversely, a low level of behavioral control would impede the performance of behavior [58]. Meanwhile, behavioral control has conceptual overlap with “complexity” in DOI, which refers to “The degree to which an innovation is perceived as relatively difficult to understand and to use” [59] (p. 238). Scholars have found farmers’ adoption behavior of organic farming is controlled by farmers’ knowledge and skills in organic farming, time availability for organic field operations, biophysical conditions of farmland; financial resources; institutional policies related to organic farming [60,61,62].

## 3. Materials and Methods

### 3.1. Study Design

This research takes a qualitative approach to understanding farmers’ motivations for adopting organic grain farming and identifying the challenges associated with their adoption process. Three reasons led to selecting a qualitative approach. Farmers’ motivations are complex, and adoption challenges may continue to be emerging and shifting. In particular, both the consumer base and the farmer base have experienced generational changes, and with them, the understanding of “organic” may have changed. A qualitative inquiry was used to elicit in-depth, rich descriptions that enable researchers to capture the complexity of issues [63,64]. We employed a qualitative study approach that focused on the “meaning, understanding, and process” of organic grain farming adoption per Merriam [63] (p. 23). We collected qualitative data through semi-structured interviews with organic farmers. Farmers were defined as the principal farm operator of each farm, and interviews were conducted on their farms. Immediate family members who influenced decisions about farm operations were also included in the interviews at the request of the farmers. The lead author conducted seventeen semi-structured, on-site (in-person) interviews from November 2017 to March 2018, with protocols for human subjects approved by the Iowa State University Institutional Review Board for both authors. The interviews were audio-recorded and transcribed with the elimination of repeated words, filler words, and speech errors.

### 3.2. Sampling Technics

This study was conducted in the state of Iowa, the largest state for both corn and soybean production in the U.S. [1]. Similar to the nation’s low adoption rate of organic farming, Iowa registered only 779 certified organic farms compared to a total of 86,104 farms [1,2]. In Iowa, 0.28% of corn production and 0.22% of soybeans production were organic [1,2]. The average size of Iowa’s organic farms (171 acres) is less than half of the state’s average farm size (355 acres) [1,2].

Both purposive and snowball sampling techniques were employed [64]. The initial interviewees were recruited by using the listservs of two non-profit farmer organizations in Iowa: the Iowa Organic Association (IOA) and the Practical Farmers of Iowa (PFI). We selected farmers who have organic grain operations. The subsequent participants were recommended by the initial interview participants and were selected to increase diverse perspectives and personal characteristics, such as age, farm size, gender, and location. We were not, however, successful in recruiting a meaningful number of female farmers. 

### 3.3. Analytical Approach

We employed a systematic analytical approach following the method merits of Corbin and Strauss [65] and Miles et al. [64]. The first cycle coding was an open coding process with constant comparisons; the second cycle coding was an axial coding process that explored the relationships between codes and categorized the codes into themes and sub-themes; the final cycle coding was a selective coding process that unified central themes that answer the research questions [64]. The coding process was performed using Nvivo 12 (QSR International, Burlington, MA, USA), a qualitative data analysis software. The second author reviewed coding categories, items within categories, and the selection of quotations several times during the iterations. The lead author presented the coding structure at local organic farmers’ meetings to collect feedback from farmer audiences and refine the coding structure to increase its face and content validity [63]. 

## 4. Results

### 4.1. Characteristics of Participants

The interview pool (Table 1) included sixteen male farmers and one female farmer, ages 35 or younger to 65 or older. Most interviewees held a college degree. At the time the interviews were conducted, fourteen farmers held organic certification, and three farmers were in the organic transition period. The participants’ organic grain operation size ranged from 80 acres to 1800 acres. Most participants owned at least a part of the farmland they operated except for three farmers, who leased all the land. Five farmers also managed livestock operations. Gender was not included in the table to protect confidentiality. Most of the participants had some level of higher education.

### 4.2. Motivations for Adopting Organic Grain Farming

This study discovered multiple motivations for farmers to adopt organic grain farming in Iowa. There are five major themes and twelve sub-themes of motivations that led farmers to the adoption of organic grain farming (Table 2).

#### 4.2.1. Self, Family, and Consumer Harm

An impetus for discontinuing conventional farming practices, and substituting organic production practices, was the claim that conventional practices caused harm. Different types of harm surfaced: harm to farmers, family members, farm workers, and consumers. Fifteen farmers deemed personal health concerns as a motivation for adopting organic farming. Eight told stories about themselves or family members who experienced health problems they attributed to exposure to synthetic agricultural chemicals used in conventional farming operations. Farmer #10 explained how health problems encountered during conventional farming led him to adopt organic farming: “There are several reasons why [I adopted organic]. The first one is my father got Parkinson’s disease. He later died from Parkinson’s disease. The insecticides that he used over the years for his farming practices—there is a direct link between those agricultural insecticides and pesticides, and development of Parkinson’s disease”.

For many farmers with children living on the farm, their health concerns were geared towards their children’s wellbeing. Farmer #09, who had three children, commented: “I don’t want to feed my kids poison. People fall all across the spectrum in terms of how they view the junk we put in our food, but I think we put poison in our food. I don’t want to feed this to my kids”.

Some farmers extended their concerns beyond themselves and their families to include consumers. Farmer #07 explained how a personal chemical incident developed into a concern for consumers’ health. Farmer #07 said: “I had one accident with 2,4-D [herbicide] one time. I got sick. I knew there were dangers and concerns about the handlers and what impact it would have on their health. Then, I just knew there was a potential for leftover residue in the food products or feed products. Then that compounds the potential problems”.

#### 4.2.2. Economic Cushion

Some farmers, who faced economic issues while managing conventional farming systems, are economically motivated to adopt organic grain production because there is the potential to receive a price premium. The concept of the price premium attracted seven farmers to adopt organic grain farming. When asked what first interested the farmers in organic farming, farmer #12 responded: “I think I heard about those $21 beans per bushel. At that time [in the 1990s], you could get $21 a bushel! I thought: man, maybe I should do that! You know, make some money. Maybe that was the real reason”.

Organic farmers have a smaller farm size on average than conventional farmers [1,2]. Small farms are often disadvantaged in competing with larger farms. Nine interviewees identified themselves as small-scale farmers and believed organic farming could help their small farms perform better in the competition. Farmer #17 said: “I transitioned because we have a small farm, and the goal was to make more money per acre so we could stay on our small farm. I got tired of all the games the chemical companies played. They were giving special prices to big farmers. They had some people that got way better, cheaper prices than we did [because] we were small”.

Because of the sluggish conventional grain markets in the years prior to the study, six farmers indicated they had to look for alternative ways to stay in farming. Farmer #03 described his rationale for converting to organic farming: “I started farming conventionally in 2011 and 2012 when corn was worth $7 and beans worth $12 to $14. Progressively, it’s gotten worse and worse. I lost money instead of making money. I knew something else was going to have to happen in order for me to stay on the farm, fully employed. So, I took the leap and started transitioning [to organic] some of my acres out of 400 acres”.

#### 4.2.3. Stewardship Legacy and Values

Land stewardship was determined as a multifaceted motivation for adopting organic grain farming. Fifteen farmers noted they chose organic farming to accomplish better land stewardship goals. 

Many of the farmers had adopted conservation practices before they started organic farming. Specifically, three farmers used no-till field management, and five farmers minimized the use of agricultural chemicals as a part of their conventional operations. Six farmers deemed land stewardship a family farm tradition. As farmer #06 said: “I feel I have a responsibility to leave it [the farm] better than I received”. Farmer #06′s father added: “I’m the second-generation farmer here. My dad before me, we have always been very conservation-minded”.

Farmers who considered conservation as a core tradition chose organic farming as a way to honor the conservation legacy and achieve land stewardship goals. Farmer #01′s wife said: “It’s also part of our overall concern for the environment and treatment of the earth. And that’s honoring conservation issues”. Farmer #10 said: “Within the conventional farming system, we were employing a lot of practices to reduce the negative impacts. Now, on the organic side, I think we’re improving the environment quite a bit by the way that we farm”.

In addition to continuing conservation traditions, regarding soil health as a success of farming also suggests another perspective of land stewardship that motivates farmers to adopt organic farming. Seven farmers stated the success of their operation relied on soil health. They saw organic farming as a way to achieve soil health. Farmer #04 identified himself as “a firm believer of soil health”, and he said: “You have got to take care of your soil health, and it can be done [in an organic farming system]”.

Organic farmers do not see a conflict between pursuing profitability and preserving natural resources. On the contrary, most stated their farm’s prosperity relies on healthy soil. Farmer #14 said: “I think maximizing our profit while preserving our natural resources, our soil. These two go hand-in-hand. Your soil life and your biology in the soil can affect your [soil] fertility”.

Four farmers indicated they had highly erodible land that had experienced serious soil loss from conventional farming management. They chose organic farming to control erosion more effectively and to improve soil health. Farmer #05 described: “I call that my field from hell! The soil had been eroding from the top ground to the bottom ground. We put in oats the first year [of organic farming], and we stopped some of that erosion. We grew clovers, and that helped too. So, not only am I thinking about raising organic grain and getting a good price for it, but I’m also thinking about what it’s doing for the soil”.

Religious beliefs also helped farmers form stewardship values and motivated some farmers to adopt organic farming. Four organic farmers identified themselves as Christian and associated their beliefs with choices regarding farming systems. These farmers stated organic farming was a way to express tenets of Christianity, follow God’s will, and trust in their faith. Farmer #11 explained: “I’m a Catholic, and so that’s important. So, this is God’s creation, and we’re to be stewards and take the best care of it. That’s an important factor in organic farming. The soil is the way God designed it. All this stuff to work is amazing, and sometimes we just have to trust. So, I think that’s a strong influence”.

#### 4.2.4. Mourning for the Losses of Biodiversity and Attachment

Another motivation for adopting organic farming is farmers’ mourning for the losses attributed to conventional farming practices. One of the losses is associated with biodiversity, and the other loss connects to a sense of attachment to the land, livestock, and social practices of an earlier era. 

Seven farmers indicated they chose organic farming because they miss farms with diversified ecologies, especially high biodiversity. When asked about why they would like to convert farmland to organic, farmer #05 answered: “There should be more biodiversity. When I came to the farm, we had red-headed woodpeckers out here. We don’t have that anymore. We used to have jackrabbits. They’re gone. It’s hard for me to say, but I know that when you think about it on all these fields where they’ve used Roundup, they destroy every plant in the field except the corn and soybeans. Well, how many insects, how many birds counted on some of those weeds, and they weren’t weeds that necessarily are competitive with the crops”.

Organic farmers believe more biodiversity will bring better ecosystem services and can solve many problems they have in crop production. For instance, Farmer #01 described: “There’s a great number of natural predatorial insects that will eat aphids; that will eat some of the nematodes in the soil. There are natural ways to combat the problems all farmers have”. Farmer #16 further explained: “So we see our health and our economy supported by biological diversity, and that’s very compatible with our quality of life. We believe that biological diversity is the answer to our problems and that diversity will give us the process”.

Six interviewees indicated they cherish the memory of the “old way of farming” that provided them with a stronger attachment to the land, and they would like to bring back the greater sense of attachment. Farmer #01 said: [I] wanted to try to bring the farm back to the way it was when farmed a long, long time ago”. Farmer #02 used to lease his land to his brother-in-law for conventional operation. He said: “While I was watching how he farmed, it just didn’t make sense that how he did that…They plant it. Co-op [agrochemical services] sprays it. They come back for harvesting. No attachment to the land at all!” When farmer #09 recalled the farm in his childhood, he said: “Without question, it’s in my blood to do things that are more closely connected to the land. When I grew up, I was raising pigs outdoors. It was row cultivating the corn instead of using chemicals. We were baling hay in the summer and putting them in for the winter to feed the animals. When I became an adult, and I started to have a family, I realized the way we farm [conventionally] today pretty much leaves out all the fun stuff. It’s like all the things that I look back on and really cherish, and see that they were valuable in my development”.

#### 4.2.5. Self-Challenging

Four farmers viewed organic farmers as a self-challenge opportunity. Farmer #12 made the organic adoption decision in the year 2000. He described his decision: “When the millennium came, so you went. But people have this feeling that it’s the millennium, and I want to do something different. And that was what I did. I transitioned all of this farm [to organic]. So, I did it in 2000”.

Being aware that organic farming would bring more challenges than conventional farming, farmer #08 said: “Everyone will tell you that’s so hard…Well, I guess I’m not afraid of hard”. Similarly, farmer #02 also said: “I always like to challenge myself. And so that’s what convinced me that even though just because not everybody in my neighborhood is farming organically, that doesn’t mean I shouldn’t do it”.

### 4.3. Challenges for Farmers to Adopt Organic Grain Farming

Farmers identified multiple challenges in the process of organic farming adoption. Five categories of challenges emerged from the interviews (Table 3). Specifically, the challenges include (1) operation and management; (2) access to organic markets; (3) information and inputs; (4) social tension, and (5) government programs.

#### 4.3.1. Challenges in Operation and Management

Thirteen farmers indicated weed control was the most common problem in raising organic grains. Conventional farming relies on synthetic herbicides, which are strictly prohibited in organic production. Weeds have been problematic for organic farmers since the early days of organic farming and are anticipated by farmers considering adoption. Farmer #07 said: “There are some really hard-to-control weeds…I knew there would be challenges there. But I didn’t realize how much trouble that would be. Particularly the tough weeds, the Canada thistle, and the giant ragweed”. 

Because of high weed pressure, farmers tend to spend more time on field operations for weed control. However, effective weed control operations (e.g., field cultivation, tillage) must be completed under good weather conditions and require an intense labor force. Therefore, time, labor, and climatic conditions turn into composite constraints in the organic farm operation. In the interview, almost all (15) farmers mentioned time, labor, and climatic constraints in their organic farming adoption process. Farmer #3 said: “Time is probably number one. Like cultivating or planting, they have to be done on a very timely basis for organic. If the weather doesn’t cooperate, you’re going to be stressed out. If I have two or three [more] people, yeah. I’ll just go over there, but it’s hard to find people who can do the work that you want to be done”. Similarly, farmer #11 also said: “It’s much, much more time-consuming…You have to do things when the weather says to do and when the weeds are the right height, and then you have to cultivate. It doesn’t matter if it’s Father’s Day or a holiday”.

Tillage practices are relied upon in organic farming systems for weed management. However, tillage can damage several aspects of soil health and can lead to erosion. Conventional conservation tillage or no-till systems, however, rely on herbicides application. Farmers who prefer to have an organic no-till system expressed they had little knowledge or experience regarding organic no-till systems. Farmer #10 said: “All those tillage operations that we were doing to control weeds make the soil compaction worse. I don’t think it’s a good idea to keep doing all this tillage to grow organic crops”. Farmer #01 has newly experimented with organic no-till farming. He said: “We are not sure if we’re going to be able to maintain organic no-till farming”, as he experienced technical issues with the system. 

Farmers use interseeding cover crops to improve soil organic matter content, manage weeds, to reduce nutrients and silt from leaving the farm. However, using interseeding cover crops introduces additional challenges for organic farming—cover crop termination. Herbicides, not allowed in organic systems, are used to suppress cover crops in conventional farming systems. Organic farmers use mechanical methods to terminate cover crops, but their use is considered to be more complicated. For instance, farmer #01 said: “We had some troubles killing the cover crops. We grew hairy vetch. It just came back. We crimped it twice and mowed it twice [until it died]”. Farmer #15 also felt frustrated with cover crop management. He said: “Every time I’ve tried [cover crops], it blew up my face. So, I’m either not using the right crop or not doing something properly”.

#### 4.3.2. Access to Organic Markets

Most farmers (11) agreed that the marketing of certified organic corn and soybeans was satisfying because the demand for organic grain has grown, and there are more organic grain buyers and brokers than before. However, the location of the markets is an issue. Eleven farmers indicated they had to sell organic grains outside the state of Iowa. The top states where they sell are Minnesota, Illinois, Wisconsin, Nebraska, and Missouri. Farther market destinations are Orgon, Arkansas, Vermont, and New York. Lack of local organic market infrastructure (e.g., organic grain elevator, organic brokers) increases marketing costs. Farmer #01 said: “We have to pay a fee for semis [trucks]. We have to ship it somewhere. Maybe to Omaha or Chicago. We have that cost. So, ten dollars all of a sudden come down to eight dollars. Just to sell it because we can’t put it in the elevator around here. There’s no elevator [for organic]”. The lack of local organic market infrastructure also impairs market efficiency. Farmer #05 shared a story: “I have this friend…took his organic grain and shipped to [an organic grain brokerage company in] Minnesota. Then he finds out his neighbor bought his organic grain for livestock from [the same company in] Minnesota. So, the same grain went away and came back again. It could have been just 12 miles [between my friend and his neighbor]”.

Because of the three-year organic transitional period, the transitional crops of the first two years are not eligible for marketing as organic grains yet need to be marketed. The marketing of organic transnational grains appeared to be a challenge. In the interview, eight farmers mentioned they had difficulties in selling their transitional organic grains with a price premium. Most of the time, farmers ended up selling their transitional corn as conventional corn without any price premium and selling the transitional soybeans into the non-GMO soybean markets with a small amount of price premium. Farmer #08 said: “Well, the challenge is how to market all your crops during the transition. I sold them on the conventional market to a local [grain] elevator, but it wasn’t easy. It was confusing”. 

Rotation crops market. Organic farmers commonly have extended crop rotations. In order to maintain the organic certification of main grain crops, the rotation crops must also be raised organically. However, marketing rotation crops, including small grains (such as oats and wheat) and hay crops, is challenging in Iowa because the crops are considered minor crops with small markets. Five farmers reported difficulties in selling hay as organic, and four farmers reported difficulties selling small grains as organic. Farmer #17 said: “Alfalfa is the hardest crop to sell because of the freight. You just can’t get enough tons on a load to justify hauling it very far. So, we sell most of our alfalfa at conventional prices”.

#### 4.3.3. Information and Inputs

Information availability related to organic farming has improved over the years. Farmer #17 said: “There was not much when we started in 2000, but there is a lot more now. They [information sources] are incredibly valuable”. However, some farmers reported information is lacking at the local level. Farmer #15 reported: “Getting local information…extension services know nothing about organic. They don’t know. A lot of times, I’ll get a call from extension [about organic farming questions]”. Farmer #01′s wife said: “Other challenges are finding resources, [such as] people really are knowledgeable about organic farming”. Farmer #01 added: “We can’t find that much locally”.

The availability of organic input showed an improvement trend. Farmers indicated organic seeds, fertilizers, and other inputs used to be less available to organic grain farmers in Iowa but have become more accessible as more organic input companies have begun doing business in Iowa. 

Nevertheless, machinery tailored to organic grain operations remains less available. Organic farmers tend to purchase more types of implements than conventional farmers, such as cultivators and rotary hoes. Preferences for tractors and implements may also be smaller in size to fit more diverse cropping systems. Finding the right type and size of equipment for organic farming is a moderate challenge for organic farmers. Farmer #02 said: “They don’t make small size equipment I need. That was kind of irritating me because I had to go more to the European equipment to find a size that was 13 feet”. Farmer #08 said: “We had a kind of a crop failure trying to grow organic peas because we didn’t have the right equipment to harvest them”. Besides sourcing the right equipment, farmers have had to learn the proper way to set up and operate the equipment. Farmer #07 said: “One year, we had really good weed control when we were raising beans. The others were not so good, mostly because of the cultivator. I just have to learn how to set the cultivator better”.

#### 4.3.4. Social Tension

Besides the technical challenges, organic farmers reported enduring social rejection in the local farming community. Even though most organic farmers exclaimed they are self-determined to adopt organic farming, social rejection is a negative experience. Farmer #07′s wife said: “We’ve got made fun of for a long time. Because in this area, people said: ‘What are you doing here? You’re going back to the horse and buggies?’ They laugh at us as much. So [my husband] just laughs with them because they couldn’t understand why you would do this”.

Because herbicides have been widely used in conventional farming operations, the “clean field” has become a gold standard for successful farming in Iowa. Visually, the organic grain farming system, together with its rotation crops, is not as “clean”. An organic field with some weeds in the rows or along the edges of rows can be labeled as a “failure” in a farming community that is dominated by conventional farming systems. Such negative social pressures can complicate the decision of farmers to consider organic farming. Farmer #01′s wife shared a story about her relatives. She reported they did not try organic farming because “they didn’t want to be embarrassed by having weeds in the field because that would make them look like bad farmers”. Farmer #01 also said he had a friend who tried organic farming only in the fields behind his grain bins to hide the attempt from other neighbors’ views. Farmer #10 described how he managed his reaction to social pressure over time: “I look around, and all of the corn and soybean fields are completely free of weeds around me. And I’m the only farmer in our township who has weedy fields. It wears on your psychology and affects your self-esteem. It really affects you quite a bit. I finally got to this point where it just doesn’t bother [me] as much”. 

Social tensions between organic farmers and conventional farmers are often exacerbated by situations where pesticides drift to organic crops. Three farmers mentioned they experienced pesticide drift incidents from neighboring conventional farming operations. Farmer #12 reported that a local pesticide applicator accidentally sprayed his organic grain transitional field. This incident caused his organic certification to be postponed. Farmer #10 assumed the tension was two-way. On the one hand, organic farmers tend to worry about the risks of pesticides drifting from operations on conventional farms. On the other hand, conventional farmers may be concerned that organic farms introduce pathogens, weed seeds, or insects to their farms because organic farmers refuse to use chemicals to control outbreaks.

#### 4.3.5. Government Programs

The federal government has established subsidy programs, such as the Environmental Quality Incentives Program (EQIP) and Conservation Reserve Program (CRP), that encourage ecological farming practices (e.g., organic farming). However, government programs are complex, and rules for organic farmers may not be well understood by the local offices’ staff who administer the programs. Three farmers indicated their local government office staff is not knowledgeable enough about how the programs work with organic farming systems. Farmer #08 said: “I had heard a little bit about this EQIP program and how it could potentially help you during the transition [to organic]. Unfortunately, my NRCS [Natural Resources Conservation Service] office staff was not knowledgeable about these programs and not helpful or timely in getting me signed up”.

Farmers criticized the complexity and redundancy of the programs’ paperwork. Farmer #12 said he had to hire a third-party consultant to complete the paperwork needed to sign up for a government program. Farmers also criticized government programs for not being sufficiently flexible to accommodate organic farming. Farmer #09 said: “I had Plan B, but I went back to Plan A because I have committed to this EQIP program. Farming in an organic rotation in itself is so complicated, and now I’ve got commitments to a program that isn’t flexible like a farmer needs to be. It’s really unjust”.

Organic farmers tend to have diverse crops and may choose an innovative cropping system, such as intercropping. Organic farmers expect to insure all types of their organic crops. However, two organic farmers complained the crop insurance they had could not provide insurance policies for their rotation crops and inter-seeding crops based on organic prices. Farmer #15 said: “I do the cover crop for alfalfa, and I put my wheat seeding, then harvest them. They will insure my corn and beans, but won’t do my seeding [crops]. Now to me, when we’re trying to control erosion, clean up water, make life better, why shouldn’t I be able to get federal crop insurance [for my seeding crops]?”

## 5. Discussion

### 5.1. Motivation Internalization

Drawing on a theoretical perspective on the self-determination continuum that describes the internalization of motivations [55,56], this study found two pathways through that farmers internalized their adoption motivations. (1) From personal safety to public health. Farmers first sought an external reward—to avoid exposure to toxic farming materials. Then extended their concerns to consumers and the general public’s health. Their motivations for adopting organic farming became to protect consumers from agri-chemicals, and they cemented the belief that organic agriculture would improve humans’ health conditions. (2) From short-term profitability to long-term viability. Some farmers initially looked for additional profitability (e.g., organic price premium) as an external reward of organic farming. However, as small farms faced more financial challenges in an increasingly competitive conventional grain market, more farmers looked for alternative farming methods to sustain their farm business. They adopted organic farming to generate more revenue without expanding their operation size and achieve their small farms’ long-term viability. To them, profitability became a pathway to long-term farm viability. The self-other valuing and its ability to translate short-term profits into long-term management goals demonstrate the motivation internalization process and provide strong anchors for an adoption rationale. 

### 5.2. Defined Values and Beliefs

Farmers are motivated to adopt organic farming when they perceive compatibility with their values and beliefs. Although many scholars recognize farmers’ value of stewardship motivates adoption decisions of organic farming [35,36,38,42], the literature seldom provides in-depth explanations. By identifying three sub-themes of stewardship and connecting them with the concept of compatibility in DOI [59], we were able to understand how farmers’ stewardship values formed as motivations to adopt organic farming. (1) Many farmers were taught conservation values from their family farms. They employed conservation practices to minimize natural resource degradation. When they discovered organic farming could improve natural resource management, many farmers embraced organic farming because they saw the practices would honor their conservation traditions. (2) Many of the farmers understood successful farming was tied to soil health. Healthier soil increased fertility and contributed to higher productivity and crop quality. (3) Some farmers linked their Christian faith to valuing and stewardship of the land. They adopted organic farming because they saw organic farming practices were compatible with religious beliefs.

We also captured farmers’ emotional reactions resulting from losses of biodiversity and strong senses of attachment to the land and to practices of prior chemical farming eras. Previous studies also found farmers’ value for biodiversity is a driver for adopting organic farming [12,32,40]. In this study, many farmers believed an ideal farming system is composed of diverse crops, insects, livestock, wildlife animals, and more. A framework common to the organic farming community states biodiversity could restore its systematic functionality and utilize ecosystem services to solve agronomic problems [66]. They believe industrialized monoculture and extensive use of chemicals have not only damaged biodiversity but have also made farmers physically and emotionally more apart from the land. Since the average size of American farms has expanded, while the number of farmers continues to decrease [67], some farmers have changed their occupations and left the land they lived and farmed. Farmers adopt organic farming because they see organic farming practices can make people better connect with the land. 

### 5.3. DOI’s Innovators?

Many farmers who adopted organic grain farming may fit Rogers’ [59] definition of high-risk, creative innovators who co-create rather than adopt. These farmers started organic farming when the majority of farmers were not well informed about organic farming. The farmers refused to follow the crowd or continue to do the same things as most other farmers. They adopted organic farming as an achievement that could differentiate them from other farmers, and they take pride in it. It may be worth observing that innovators may not be the individuals who make sufficient funds to stay in the conventional community, and they may not be the “opinion leaders” whom others follow. The farmers were not afraid of failures and challenges, even though they had to bear negative social pressure and faced technical difficulties. These farmers’ venturesome characteristics suggest farmers have a high level of autonomy, which further explains why farmers sought self-challenges in their adoption process of organic farming.

### 5.4. Evolving Challenges for Organic Grain Farming

This study identified multiple challenges for producers who raise organic grains in Iowa. There are five main areas of challenges, to varying degrees, that impeded farmers’ adoption of organic grain farming. From TPB’s behavioral control perspective [57], our findings suggest farmers’ organic farming adoption behavior is controlled by farmers’ skills in the area of organic farming operation and management, accessibility of organic grains market, availability of information and inputs relevant to organic farming, negative social pressures, and support through government programs. These challenges also empirically illustrate the complexity of organic agriculture framed by DOI [13,58]. By reconnecting the interview results with relevant literature, we discuss below how the challenges evolved and impeded farmers from adopting organic grain farming.

#### 5.4.1. The Derivative Problems of Weed Control in Organic Farming

Most of the operation and management challenges noted by grain farmers are related to weeds, a longstanding issue across years, regions, and commodities [15,16,34,42,45]. This study refined the observations. Weed control has been a two-fold problem for organic grain farmers. One is about the technical difficulties of weed control. Additionally, weed control has caused labor and time management issues. Herbicide application reduced the need for labor and simplified field management practices for conventional farmers. However, organic farmers must retain a labor force, spend more time on-field operations, and seek multiple implements for diverse crops in the growing season to successfully manage weed pressure. 

In this study, unlike prior studies, neither pest control nor disease control was stated as a noteworthy problem by organic grain farmers. This may be because previous studies focused on organic fruit and vegetable producers or livestock producers [15,16,35,41,42,44,45]. The production of fruit, vegetables, and livestock is generally more susceptible to pests and diseases than grain production. 

Organic farmers commonly use mechanical methods to control weeds and manage cover crops, such as tillage or cultivation. However, more frequent tillage operations in organic farming systems have been criticized for contributing to soil compaction, crusting, erosion, and reducing soil organic matter content [68,69]. Although crop rotations with small grains and hay crops somewhat offset tillage drawbacks, tillage operations are not aligned with organic farmers’ values on natural resource conservation and soil health. Therefore, farmers are inclined to organic no-till farming systems. Research on organic no-till systems has been conducted [70,71], but the no-till techniques that fit Iowa’s landscape and climate are still being refined through on-farm research at the time of this study [72]. 

Farmers discussed cover crop management as a challenge. This was reported less in previous studies. With the federal government’s financial support, the use of cover crops in the U.S. has become more widely accepted [73]. From 2012 to 2017, cover crop usage surged in Iowa. The acres of cover crops increased 156% over these five years [74]. Farmers use cover crops for better soil and water conservation, such as reducing soil erosion and maintaining nitrate-nitrogen in the field [73]. Organic farmers use cover crops as a multifunctional management tool to suppress weeds, fix nitrogen, diversify the cropping system, and increase soil organic matter [75]. Roesch-McNally et al. [76] found in the U.S. Midwest, there are structural obstacles and technical difficulties for farmers to use cover crops. Therefore, how to appropriately grow cover crops to better improve soil fertility and how to effectively terminate cover crops without herbicides drew organic grain farmers’ attention. Thus, they perceived this as a challenge.

#### 5.4.2. Localized and Specialized Marketing Difficulties

This article began by noting the high demand for organic grains, but for farmers, a key problem has been market access. Compared with earlier literature [12,15,34,36,41], the challenges of marketing organic grain seemed to improve to some extent, along with an increase in consumer demand. However, at the local level, Iowa’s organic grain market infrastructure (principally grain elevators) is less abundant than the infrastructure for conventional grain. Many farmers may contract to sell their grains to buyers from other states, but the cost of freight is part of the contract. Farmers may benefit from contracts with in-state organic grain buyers and brokers. In addition to offering attractive contacts, local organic grain brokers may better connect with the local organic livestock producers and organic food processors on behalf of grain producers.

The organic transition period poses financial risks because, during the transitional period, farmers are required to raise crops organically, but they cannot sell their crops as organic. This finding confirms earlier results from studies of financial challenge phases in organic farming [12,16,45]. To offset financial challenges, organic grain farmers in Iowa sell their transitional grains to non-GMO markets, although the price is not typically as high as the full organic price premium. Farmers also strategize their transition plan based on risk preference, operation and management skills, availability of markets, and the biophysical conditions of the land.

Organic farmers grow hay crops (e.g., alfalfa and clover hay) and small grains (e.g., oats, wheat, barley, rye) as rotation crops help to break the growth cycles of weeds, disease, and insects [77]. Compared with the main row crops (corn and soybean), the deeper and larger rooting system of hay crops and small grains increases soil microorganisms and improves soil organic matter content [78]. However, it can be a challenge for many Iowa organic farmers to market their organic hay and small grains they do not use on their farms (i.e., for livestock). In Iowa, most grain production is separate from producers who use hay—the organic dairy producers clustered in the northeastern and southeastern areas of the state. Organic farmers from other areas have difficulties in selling their hay to organic livestock farmers because hay is particularly heavy and bulky, with high freight costs.

Marketing small grains is a challenge in Iowa because the traditional market outlet of small grains has dropped with the consolidation and vertical integration of livestock production; yet, the new specialized market opportunities (food-grade small grains, cover crop seeds, and peer-to-peer exchanges network) is still under development [79]. Many organic farmers target food companies as the market for their organic small grains, but food-grade organic small grains require a higher quality standard that needs free of weeds, insects, and mold. Farmers often need to take extra steps and sometimes need additional equipment to harvest, clean, and store these small grains [80].

#### 5.4.3. Local Information Gap

Information deficiency was a major challenge for early organic farmers across regions [15,34,42,45]. Information gaps included marketing channels, farming techniques, input sources, and certification procedures. Nowadays, most organic farmers locate most information through multiple information channels, including the internet. However, many of these farmers expressed frustration with difficulties in locating relevant information about key resources at the local levels, specifically within the county. One possible reason is county-level extension educators’ lack of knowledge and experience in organic (grain) farming. Even though many land-grant universities have established organic agriculture programs (such as Iowa State University Organic Agriculture Program), the number of organic experts is limited, and farmers remark that the local educators are unable to provide sufficient extension services to organic grain farmers in the state. 

#### 5.4.4. Machinery Lags behind Inputs

Previous studies found organic farmers cannot always locate inputs for organic farming, including organic seed, manure, organic pesticides, and specialized equipment for organic production [15,16,41,42,45]. Our findings suggest the availability of most organic inputs has improved because more organic input businesses have started to operate in Iowa. The IOA [81] compiled a directory of organic suppliers listing more than two hundred companies that sell organic inputs in Iowa. However, farmers still have difficulties in finding machinery specialized in organic grain operation. Three reasons can cause this. (1) Organic farmers need to source a wider variety of specialized equipment to fit diverse cropping systems. (2) Organic farmers tend to be more sensitive to climatic conditions. Different types of equipment are needed to fit the changing weather conditions. (3) Organic farmers prefer to use smaller size equipment because organic farmers have smaller farming scales and are often divided into multiple plots for rotation purposes, but today’s major agricultural machinery manufacturers tend to make more large-scale farm equipment.

#### 5.4.5. Enduring or Easing Social Tension?

Organic farming has been poorly accepted in the American rural community [35,36,41]. The interviews identified social tension between organic and conventional farmers. Farmers who adopted organic farming described social pressure from their neighbor farmers and extended family members who have conventional farm operations. There are mainly two factors that lead to social tension. First, conventional farmers and organic farmers have different definitions of successful farming. Conventional farmers see large-scale, highly industrialized, clean monocultural fields as successful farming. In contrast, organic farmers view success as a smaller farming size, healthier soil, and more diverse crops [60]. The second factor is that farmers on both sides are worried that their counterparts will bring potentially harmful risks to their farms. Specifically, organic farmers are concerned about pesticide drift from conventional farms, and conventional farmers are worried that organic farms would bring weed seeds, pests, or pathogens to their farmland or file a lawsuit when pesticide drift occurs. Though TPB theorizes subjective norm positively motivates behavioral intention [57,58], these findings suggest negative social tension toward organic agriculture discouraged farmers who may consider adopting organic grain farming. 

#### 5.4.6. Program Access Issues

Compared with earlier studies [34,36,41], federal government policies toward organic farming have become more favorable through programs, including EQIP, a certification cost-share program, and more friendly insurance provisions for organic crops [4]. However, at the policy implementation stage, three problems are identified in this study. (1) Government office staff lack the expertise to guide farmers on how to enroll their organic operations into different programs. (2) The programs’ application procedures require complicated and extensive paperwork beyond some farmers’ capabilities. (3) Some programs lack adequate flexibility to better accommodate diverse and innovative cropping systems for organic farming. The 2018 Farm Bill provides more incentives for organic farming conversion, more accommodating insurance policies, and expands funding to support organic farming research and extension education [29]. These identified problems may be eased with further implementation of the 2018 Farm Bill.

## 6. Conclusions, Implication, and Limitations 

This study aimed to address the gaps in the literature that lack an understanding of the motivation and challenges of U.S. Midwest farmers in adopting organic grain farming by collecting empirical data from grain producers in farmers in Iowa through 17 individual, in-person interviews. The first part of this study identified five major factors that motivate farmers to adopt organic grain farming. Farmers presented both extrinsic and intrinsic motivations to adopt organic grain farming. For selected elements, the combination of extrinsic and intrinsic motivations likely provides enduring drivers. In addition, compatibility with three types of values and beliefs has motivated farmers to adopt organic grain farming. Meanwhile, personal characteristics of self-challenge and risk-taking also motivated organic grain adoption. The second part of this study identified five areas of challenges that farmers reported hindered them from readily adopting or persisting in organic grain farming. Some findings confirmed earlier conclusions in the literature, and some were new or appeared specific to grain farming in Iowa. Specifically, (1) As farmers incorporate new practices such as cover crops and organic no-till systems, they are faced with a series of weed management challenges related to implementing these practices. (2) Marketing challenges remain with transitional and rotational crops. (3) There is still a lack of organic farming information at a local level. (4) Availability and accessibility of organic farming machinery lag behind the development of organic inputs. (5) Social tensions exist between organic farmers and their neighboring conventional farmers. (6) Government programs are too complex and rigid for organic farmers.

The findings and conclusions of this study provided implications for extension education program development and agricultural policy making. (1) Agricultural extension educators and organic specialists are recommended to approach farmers by presenting what problems organic farming systems can help to solve and what long-term management goals organic farming systems can help to achieve, then facilitate farmers to discover how organic agriculture aligns with their values and belief systems. The education programs need to strategize their teaching topics to progressively help farmers overcome the learning curves: Prioritize basic organic agronomic measures for beginning organic farmers and expand the topics to complex and emerging issues for experienced organic farmers. (2) To more efficiently increase organic adoption, both policy and educational programs of organic agriculture may foremost target farmers who have already participated in the Natural Resources Conservation Service’s conservation programs and synergize conservation practices with organic farming. (3) Collaborate with organic farmers to co-create new methods, machinery, and techniques of organic farming by conducting more on-farm research; empower farmers to co-develop related policies by using participatory policy-making approaches. (4) To reduce social tensions between organic and conventional farmers, we recommend that organic experts use mainstream agricultural communication channels to provide more publications on the general rules and principles of organic farming. Helping local conventional farmers become more informed about this growing alternative farming system may change their stereotypes of organic farmers and lessen conflicts caused by different farm operational methods. (5) We recommend that organic specialists and extension educators develop more programs focused on organic crop marketing by inviting local organic grain brokers and experienced farmers to share their experiences and strategies related to organic grain marketing. We also recommend that policymakers strengthen marketing assistance programs for transitional crops to reduce farmers’ financial difficulty during the transitional period.

Though this study yielded rich empirical data and provided in-depth answers to the two research questions, the findings, at least two limitations, may be raised with the nature of this qualitative study. First, given the small sample size of this study, the conclusions cannot be directly generalized to a broad population. The concept of transferability of findings replaces the idea of generalizability for a qualitative study such as this. Findings will be applicable mainly to situations where organic grain operators are an audience, particularly in the Midwest, but likely also in other regions of the US and Canada where grains are raised. In addition, we identified important farmers’ motivations and major challenges. However, it was noticed that as market conditions changed, organic farmers’ motivations and challenges continued to evolve. Therefore, it is recommended more empirical studies are needed to examine the dynamics of motivations and challenges along with any significant changes in policies or environmental conditions that may occur in the future.

## Figures and Tables

**Table 1:** Participant’s Demographics and Farm Structural Characteristics.

ID	Age Group	Year of 1st Certificate	Organic Grain Operation Acres	Percent of Land Owned	Livestock Operation	Education Level
#01 *	65 or older	2018	80	100%	No	Graduate
#02	55–64	2001	100	100%	No	4-year College
#03 *	35 or younger	2018	80	0%	Yes	Graduate
#04	65 or older	2015	153	0%	No	Less than GED or High School
#05	65 or older	2017	137	80%	No	4-year College
#06	45–54	1998	450	60%	Yes	4-year College
#07	55–64	2009	228	100%	No	4-year College
#08	45–54	2014	250	100%	No	Graduate
#09 *	35–44	2018	40	10%	No	Graduate
#10	55–64	2008	130	100%	No	4-year College
#11	36–44	2000	372	40%	Yes	4-year College
#12	65 or older	1998	260	40%	No	4-year College
#13	35–44	2007	120	0%	No	Graduate
#14	55–64	1998	1800	90%	No	4-year College
#15	65 or older	1984	700	100%	No	GED
#16	55–64	1998	140	100%	Yes	2-year College
#17	65 or older	2000	200	60%	Yes	4-year College

*Note.* * The farmers were in organic transition when the interviews were conducted in 2017, and they were certified in 2018.

**Table 2:** Themes and Sub-themes of Motivations to Adopt Organic Grain Farming.

Themes of Motivations	Sub-Themes		
Self, family, and consumer harm	Health problems related to chemicals	Concerns for personal and family health	Concerns for public and consumer health
Economic cushion	Profitability	Sluggish conventional grain market	Small farm viability
Stewardship legacy and values	Conservation tradition	Soil health as success	Religious beliefs
Mourning for the loss	Values of biodiversity	Attachment to the land	
Self-challenging	Self-challenge		

**Table 3:** Themes and Sub-themes of Challenges of Organic Grain Farming Adoption.

Themes of Challenges	Sub-Themes			
Operation and management	Weed control	Time, labor, and weather	No-till systems	Cover crop management
Access to organic markets	Local market infrastructure	Transitional crops marketing	Rotation crops marketing	
Information and inputs	Local extension services	Machinery for organic		
Social tension	Negative interactions with conventional farmers	Different standards for successful farming	Pesticide drift and uncontrolled pests	
Government programs	Office staff lack knowledge	Complexity of government programs	Inflexible government programs	

## Data Availability

The data that support the findings of this study are available on request from the corresponding author. The full data are not publicly available because they contain information that could compromise research participant privacy/consent.
